# Pilot Study of Lemborexant for Insomnia in Cancer Patients with Delirium

**DOI:** 10.1089/jpm.2021.0509

**Published:** 2022-04-20

**Authors:** Tatsuto Terada, Takatoshi Hirayama, Ryoichi Sadahiro, Saho Wada, Rika Nakahara, Hiromichi Matsuoka

**Affiliations:** Department of Psycho-Oncology, National Cancer Center Hospital, Tokyo, Japan.

**Keywords:** cancer patients, delirium, insomnia, lemborexant, orexin receptor antagonist

## Abstract

Delirium occurs very frequently in cancer patients. Insomnia is a symptom of delirium. Lemborexant is a drug that regulates sleep–wake rhythms without causing extrapyramidal symptoms. Based on its ability to improve sleep, lemborexant is expected to have efficacy for insomnia with delirium. The purpose of this study was to determine the efficacy of lemborexant for insomnia in cancer patients with delirium. A retrospective observational study was conducted between July 2020 and February 2021. Fourteen patients (six females; mean age,69 years) were included. Lemborexant was effective in 11 of 14 (78.6%) patients. Of 14 patients, 10 had hyperactive delirium. Lemborexant might have similar efficacy for insomnia with and without delirium when compared with previous studies. The efficacy rate of lemborexant was 70% for patients with insomnia and hyperactive delirium. This study might lead to dose reductions of antipsychotic medications and fewer extrapyramidal symptoms in cancer patients with delirium.

## Introduction

Delirium occurs in ∼40%^1^ of hospitalized cancer patients requiring a palliative care consultation and in 90%^2^ of patients at the end of life. Disturbance of sleep–wake rhythm (DSWR) occurs in 97%^3^ of patients with delirium. DSWR, which includes insomnia, is considered a symptom of delirium in the World Health Organization's International Statistical Classification of Diseases and Related Health Problems, 11th Revision.^4^

The relationship between delirium and insomnia involves several mechanisms. DSWR increases levels of inflammatory cytokines,^5^ which are correlated with delirium.^6,7^ Inflammatory cytokines cause decreased rapid eye movement sleep (REMS).^8^ Impaired REMS is associated with delirium.^9^

Benzodiazepines and Z-drugs, drugs whose names often begin with Z, are commonly used for insomnia. They are risk factors for delirium.^2^ These drugs decrease REMS^10^ and should be avoided in patients with insomnia at high risk for delirium, including cancer patients. Treatment of insomnia that does not rely on benzodiazepines or Z-drugs and does not cause delirium is needed.

Lemborexant is a dual orexin receptor antagonist that works on orexin 1 receptor (OX1R) and orexin 2 receptor (OX2R). Lemborexant is indicated for the treatment of insomnia. It has been shown to be safer and more efficacious than zolpidem, the most frequently used sleep medication, in a global phase III study.^11^ Lemborexant promotes sleep without decreasing the amount of REMS.^12^ Lemborexant might be effective for insomnia without worsening delirium, but there are no studies evaluating this hypothesis. The purpose of this study was to determine whether lemborexant could decrease insomnia in cancer patients with delirium.

## Methods

This study involved a retrospective chart review to evaluate the efficacy of lemborexant for insomnia in cancer patients with delirium. It was conducted at the National Cancer Center Hospital (NCCH) in Tokyo, Japan.

Eligibility criteria included (1) being diagnosed cancer and delirium, (2) being managed with scheduled antipsychotics for delirium by a psycho-oncologist, (3) age of 20 years old or older, (4) being prescribed lemborexant for insomnia from July 2020 to February 2021, (5) insufficient effect on delirium (i.e., having insomnia and other symptoms that meet diagnostic criterion for delirium) with antipsychotics before scheduled doses of lemborexant, and (6) availability of documented patient's reported outcome (PRO) regarding the efficacy for insomnia.

We collected the following data: background (age, gender, cancer type, insomnia type, and delirium type), baseline laboratory data before administration, PRO (effective or ineffective), psychotropic drug use, lemborexant dose, lemborexant administration (switch, switching from a previously administered psychotropic drug, or add, as an addition to psychotropic drugs), and adverse events (AEs).

The diagnosis and classification of delirium in the medical records were made by psycho-oncologists according to the American Psychiatric Association's *Diagnostic and Statistical Manual of Mental Disorders* (fifth edition, DSM-5).^13^ For this study, another psycho-oncologist confirmed the diagnosis and classification of delirium based on medical records according to DSM-5 criteria. All patients were receiving antipsychotic medications.

Evaluations of PRO were determined by two psycho-oncologists and a psychiatric nurse specialist who used medical records to identify the best PRO within five days of the first prescription of lemborexant for each patient. To minimize the effect of decreasing delirium over time, we matched the duration of drug administration for delirium reported in previous studies.^14^ PRO was classified as effective if the patient made statements such as “I slept well.” PRO was classified as ineffective if there was no patient assessment of “I slept well” during the observation period, the patient continued to complain of insomnia, additional psychotropic medication was used, or the patient clearly had insomnia based on nursing records. If the three experts disagreed, PRO was classified as ineffective.

This study was approved by our institutional review board. The requirement for informed consent was waived due to the retrospective design. Opt-out information was published on the NCCH website.

### Analysis

Descriptive statistics were used to analyze the demographic data and compare the characteristics of patients in the effective and ineffective groups. Fisher's exact test was used to assess the determinants of efficacy of lemborexant for insomnia in cancer patients with delirium. Statistical analysis was performed using JMP14 (SAS Institute, Cary, NC, USA). Statistical significance was set at *p* < 0.05.

## Results

We included 14 cancer patients (6 females) in this retrospective analysis. Age ranged from 53 to 83 years (mean, 69.0 ± 11.4 years) (Table 1). Five patients had hepatobiliary pancreas cancer (35.7%) and three had hematological cancer (21.4%). Seven patients had difficulty falling asleep, three had difficulty maintaining sleep, one had both, and three were unsure. There were 10 patients (71.4%) with hyperactive delirium. The most common psychotropic drugs were chlorpromazine in six patients (42.9%), haloperidol in five patients (35.7%), and trazodone in four patients (28.6%). Each drug was used in combination.

**Table 1.** Clinical Characteristics of the Study Patients

Patient	Age (years)	Gender	Cancer type	Insomnia type^a^	Delirium type	Therapy type	Psychotropic drug	Dose (mg/day)		Efficacy			AE
								Starting	Maximum	overall evaluation	PRO	Additional use^b^	
1	53	F	HM	Maintain	Hypo	Add	Trazodone, haloperidol	5	5	Effective	Good	No	No
2	55	F	HBP	Initiate	Hyper	Add	Asenapine	5	5	Effective	Good	No	No
3	82	M	Lung	Maintain	Hypo	Switch	Trazodone, risperidone	5	5	Effective	Good	No	No
4	75	F	Lung	Initiate	Hyper	Switch	Zolpidem, suvorexant, olanzapine	5	5	Effective	Good	No	No
5	83	M	Urological	N/A	Hypo	Add	Haloperidol	5	5	Effective	Good	No	No
6	67	F	HM	N/A	Hypo	Switch	Hydroxyzine, risperidone	2.5	5	Effective	Good	No	No
7	61	F	HM	N/A	Hyper	Add	Hydroxyzine, asenapine, haloperidol	2.5	2.5	Ineffective	Not good	No	No
8	79	M	HBP	Initiate	Hyper	Switch	Zolpidem, chlorpromazine, quetiapine, Haloperidol	2.5	5	Ineffective	Good	Yes	Liver dysfunction
9	78	M	HBP	Initiate	Hyper	Switch	Chlorpromazine	5	5	Ineffective	Not good	No	No
10	55	M	Colorectal	Initiate	Hyper	Add	Zolpidem, quetiapine	5	5	Effective	Good	No	No
11	79	M	HBP	Initiate	Hyper	Switch	Trazodone, chlorpromazine	5	5	Effective	Good	No	No
12	73	M	Head/neck	Initiate/maintain	Hyper	Switch	Chlorpromazine, quetiapine	5	5	Effective	Good	No	No
13	53	M	HBP	Initiate	Hyper	Add	Hydroxyzine, chlorpromazine, haloperidol	5	5	Effective	Good	No	No
14	73	F	Sarcoma	Maintain	Hyper	Add	Trazodone, etizolam, chlorpromazine	5	5	Effective	Good	No	No

^a^ Based on symptoms reported by the patient and nursing observations in the medical record.

^b^ Refers to whether psychotropic medication was added or not.

HBP, hepatobiliary or pancreatic; HM, hematological; hyper, hyperactive type; hypo, hypoactive type; N/A, not available; PRO, patient reported outcome.

Lemborexant was administered in seven patients as a “switch” and seven patients as an “add.” It was effective in five of seven “switch” cases and in six of seven “add” cases. The distribution of delirium subtype was the same in both groups. Lemborexant was initiated at a dose between 2.5 and 5 mg per day. The maximum maintenance dose was 5 mg per day.

For 11 patients (78.6%), lemborexant was evaluated as effective; it was evaluated as ineffective for three patients (21.4%). The patient characteristics of the effective and ineffective groups were not significantly different (Table 2).

**Table 2.** Descriptive Statistics of Patients' Clinical Characteristics by Group

	Effective		Ineffective		Total	*p*
Age						
≥70 Years	6	42.9%	2	14.3%	8	1.000
<70 Years	5	35.7%	1	7.1%	6	
Gender						
Male	6	42.9%	2	14.3%	8	1.000
Female	5	35.7%	1	7.1%	6	
Cancer type						
HBP	3	21.4%	2	14.3%	5	
HM	2	14.3%	1	7.1%	3	
Lung	2	14.3%	0	0.0%	2	
Other	3	21.4%	0	0.0%	3	
Insomnia type						
Maintain	4	28.6%	0	0.0%	4	
Initiate	6	42.9%	2	14.3%	8	
Delirium type						
Hyperactive	7	50.0%	3	21.4%	10	0.506
Hypoactive	4	28.6%	0	0.0%	4	
Method						
Add	6	42.9%	1	7.1%	7	1.000
Switch	5	35.7%	2	14.3%	7	
Psychotropic drug^a^						
Haloperidol	3	21.4%	2	14.3%	4	
Risperidone	2	14.3%	0	0.0%	2	
Chlorpromazine	5	35.7%	1	7.1%	6	
Olanzapine	1	7.1%	0	0.0%	1	
Asenapine	1	7.1%	1	7.1%	2	
Quetiapine	3	21.4%	0	0.0%	3	
Zolpidem	3	21.4%	0	0.0%	3	
Suvorexant	1	7.1%	0	0.0%	1	
Trazodone	4	28.6%	0	0.0%	4	
Hydroxyzine	2	14.3%	1	7.1%	3	
AE						
Yes	0	0.0%	1	7.1%	1	0.214
No	11	78.6%	2	14.3%	13	
Laboratory data at baseline						
Alb ≥ mean^b^	6	42.9%	0	0.0%	6	0.209
	5	35.7%	3	21.4%	8	
AST ≥ mean^b^	7	50.0%	1	7.1%	8	0.539
	4	28.6%	2	14.3%	6	
ALT ≥ mean^b^	4	28.6%	1	7.1%	5	1.000
	7	50.0%	0	0.0%	7	
ALP ≥ mean^b^	2	14.3%	1	7.1%	3	1.000
	9	64.3%	2	14.3%	11	
Bil ≥mean^b^	5	35.7%	1	7.1%	6	1.000
	6	42.9%	2	14.3%	8	
Cre ≥ mean^b^	6	42.9%	0	0.0%	6	0.209
	5	35.7%	3	21.4%	8	
eGFR ≥60 mL/min/1.73 m^2^	7	50.0%	3	21.4%	10	0.506
	4	28.6%	0	0.0%	4	

^a^ Each drug was used in combination with other psychotropic drugs.

^b^ Mean value was calculated from 14 patients' values. Mean Alb value = 2.78 g/dL. Mean AST value = 25.4 IU/L. Mean ALT value = 26.2 IU/L. Mean ALP value = 142.3 IU/L. Mean Bil value = 0.51 mg/dL. Mean Cre value = 0.73 mg/dL.

Alb, serum albumin; ALP, alkaline phosphatase; ALT, alanine transaminase; AST, aspartate aminotransferase; Bil, total bilirubin; Cre, creatinine; eGFR, estimated glomerular filtration rate; HBP, hepatobiliary or pancreatic; HM, hematological.

One patient (7.1%) discontinued lemborexant because of liver dysfunction. No other AEs occurred, including oversedation or extrapyramidal symptoms.

## Discussion

In this study, we demonstrated that lemborexant was effective for insomnia in 11 of 14 cancer patients with delirium (78.6%). The efficacy rate of lemborexant 5 mg per day in adult patients with insomnia (mean age, 63.7 ± 6.8 years) in a previous study^11^ was 82.0%; the patient population and efficacy rate of the two studies seemed similar. Lemborexant might have similar efficacy in insomnia with and without delirium. Lemborexant had an efficacy rate of 70.0% for treating insomnia with hyperactive delirium. Improving sleep–wake rhythms in patients with delirium using lemborexant might lead to decreased delirium.

Antipsychotics are commonly used to treat delirium.^15^ However, several studies have suggested that antipsychotics are not beneficial; their use has been questioned.^16^ Antipsychotics have problems such as extrapyramidal symptoms and oversedation. Agitation is a common problem in delirium. When antipsychotic doses are increased to treat agitation, there is a risk of worsening extrapyramidal symptoms. Lemborexant is a drug that contributes to appropriate sleep–wake rhythms without extrapyramidal symptoms. This study might lead to reductions in antipsychotic dosage and extrapyramidal symptoms. Lemborexant might be useful when it is difficult to increase the dose of antipsychotics due to extrapyramidal symptoms or when a dose reduction is necessary.

The orexin system provides a reason to expect that lemborexant can be effective for insomnia without worsening delirium. The orexin system has recently become a focus of attention in the regulation of sleep–wake rhythms. OX2R is known to be more important than OX1R in the regulation of sleep–wake rhythms.^17^ Orexin receptors include OX1R and OX2R. Orexin-A and orexin-B are known agonists. OX1R has a high affinity for orexin-A, and OX2R has a similar affinity for both orexin-A and orexin-B.^18^ A previous study^19^ showed that orexin-A is elevated in the serum of patients with agitated delirium. Another recent study^20^ showed that OX2R stimulation is associated with accelerated aggression in rodents. These findings suggest that orexin receptor antagonists might have efficacy for agitation, and that OX2R is particularly important. Lemborexant has a high affinity for OX2R.

Lemborexant was ineffective for three patients in this study. One patient requested discontinuation of lemborexant, another patient discontinued lemborexant due to liver dysfunction, and the third patient had an unspecified reason. The patient who experienced liver dysfunction had pancreatic cancer and was treated under the policy of best supportive care only; thus, liver dysfunction could have been due to the progression of the primary disease.

This study has several limitations. First, there are no clear criteria for dosing lemborexant. Patients for whom lemborexant was classified as ineffective did not reach the maximum dose of 10 mg per day. There is not enough information to determine whether the dose was insufficient or lemborexant was ineffective. Second, the state of delirium could not be assessed quantitatively using criteria such as the Memorial Delirium Assessment Scale or the Richmond Agitation-Sedation Scale; delirium could only be assessed according to DSM-5 criteria.

Third, lemborexant is efficacious in combination with other psychotropic drugs. This study might not be able to show the efficacy of lemborexant monotherapy. Fourth, due to the short observation period, the assessment of AEs during long-term administration might have been insufficient. Finally, this study was a single-center retrospective study with a small number of subjects. It might be difficult to generalize these results.

This study is the first to suggest that lemborexant is efficacious for treating insomnia in cancer patients with delirium. Despite some limitations, our findings suggest that lemborexant can be expected to have efficacy for insomnia in such patients. We hoped that decreased insomnia would be effective in decreasing delirium. We will conduct a prospective study to explore the efficacy of lemborexant as a novel therapy for delirium.
